# *In vitro* characterization of GSK2556286 in combination with bedaquiline and pretomanid against *Mycobacterium tuberculosis*

**DOI:** 10.1128/aac.00260-26

**Published:** 2026-07-13

**Authors:** Marie Sylvianne Rabodoarivelo, Jordana Galizia, Deborah Recchia, Alessandro Stamilla, Michael Dal Molin, Eik Hoffmann, Cyril Gaudin, Maxime Eveque, Dominique Ndjogou, Rebeca Bailo, Albin A. M. Leding, Ulrika S. H. Simonsson, Olalla Sanz, Elena Jiménez, Robert H. Bates, Giulia Degiacomi, Ainhoa Lucía, Nicolas Willand, Alain Baulard, Jan Rybniker, Maria Rosalia Pasca, Santiago Ramón-García, on behalf of the ERA4TB consortium

**Affiliations:** 1 Department of Microbiology, Pediatrics, Radiology and Public Health, Faculty of Medicine, University of Zaragoza 189151, Zaragoza, Spain; 2 Department of Biology and Biotechnology Lazzaro Spallanzani, University of Pavia 111209, Pavia, Italy; 3 Division of Infectious Diseases, Department I of Internal Medicine, University of Cologne 14309, Cologne, Germany; 4 Faculty of Medicine, Center for Molecular Medicine Cologne (CMMC), University of Cologne 61059, Cologne, Germany; 5 University of Lille, CNRS, Inserm, CHU Lille, Institut Pasteur de Lille, U1019-UMR 8204, Centre d'Infection et d'Immunité de Lille (CIIL) 56099, Lille, France; 6 University of Lille, Inserm, Institut Pasteur de Lille, U1177-Drugs and Molecules for Living Systems 173548 , Lille, France; 7 Department of Pharmaceutical Biosciences, Uppsala University 8097, Uppsala, Sweden; 8 Global Health Medicines R&D, GlaxoSmithKline GSK, Tres Cantos, Madrid, Spain; 9 Spanish Network for Research on Respiratory Diseases (CIBERES), Carlos III Health Institute 38176, Madrid, Spain; 10 German Center for Infection Research (DZIF), Partner Site Bonn-Cologne 459706 , Cologne, Germany; 11 Fondazione IRCCS Policlinico San Matteo 18631 , Pavia, Italy; 12 Research and Development Agency of Aragon Foundation (Fundación ARAID) 203260, Zaragoza, Spain; Queen Mary University of London, London, United Kingdom

**Keywords:** tuberculosis, regimen, GSK2556286 (GSK286), dose-response time-kill assay, pharmacodynamics

## Abstract

GSK2556286 (GSK286) is a novel drug candidate with a cholesterol-dependent mode of action that has shown promising *in vivo* potential to improve tuberculosis treatment when combined with bedaquiline (B) and pretomanid (Pa). This study explored the *in vitro* activity of GSK286 alone and in combination with B and Pa, using extracellular and intracellular dose-response time-kill assays against different *Mycobacterium tuberculosis* (Mtb) strains. GSK286 demonstrated media-specific activity, inhibiting Mtb growth only in cholesterol- and propionate-containing media, with slight activity against both extracellular and intracellular Mtb. The Mtb strain overexpressing Mtb adenylyl cyclase *Rv1625c* exhibited higher susceptibility to GSK286, whereas the *Rv1625c* mutant was more resistant. This confirms GSK286’s mechanism of action linked to *Rv1625c*. GSK286 combined with BPa was bactericidal, although the main driver of this interaction was observed between GSK286 and B, with potency comparable to that of the triple BPa + GSK286 regimen, both extracellularly and intracellularly. These findings suggest that GSK286 may be a promising companion to B, with or without Pa, particularly under conditions with a sub-optimal B dose, where the GSK286 + B interaction was more clearly observed. These studies offer new insights into this drug combination to guide future research toward better tuberculosis treatments.

## INTRODUCTION

Tuberculosis (TB) continues to be a global public health challenge, with approximately 1.25 million deaths each year worldwide (1). The emergence of *Mycobacterium tuberculosis* (Mtb) drug-resistant strains further complicates the fight against TB, requiring the development of new drugs and treatment regimens to improve cure rates. The World Health Organization has recently recommended a novel treatment regimen incorporating bedaquiline (BDQ, B), pretomanid (PTD, Pa), and linezolid (LZD, L) (BPaL) that ensures optimal efficacy against Mtb multi-drug- or rifampicin-resistant (MDR/RR) strains within 6 months of treatment (2). Nevertheless, this new therapeutic option is already encountering several limitations. Linezolid may cause peripheral neuropathies in a dose- and duration-dependent manner (3). The activity of pretomanid appears to vary by *Mycobacterium tuberculosis* complex (MTBC) lineage, with lineage 1 (L1) intrinsically less susceptible than other major lineages (4, 5). This raises the question of whether the BPaL regimen remains equally effective in patients infected with this lineage, which accounts for the largest absolute number of TB cases worldwide among all lineages (approximately 28% of the global TB cases) (6, 7).

GSK2556286 (GSK286) was identified in high-throughput intracellular screenings (8–10) and has a novel mechanism of action. It inhibits Mtb growth both intracellularly within human macrophages and extracellularly in the presence of cholesterol as the only carbon source. *In vivo* studies using an Mtb-infected BALB/c mouse model demonstrated that the administration of GSK286 in combination with BPa yielded similar efficacy outcomes as the BPaL regimen, making GSK286 a promising companion drug in new regimens for treating drug-susceptible and drug-resistant TB (10).

In this study, we conducted an *in vitro* assessment of the GSK286 molecule in combination with the backbone BPa. This investigation explored the impact of drug concentration modulation on the overall interaction, aiming to identify optimized concentrations for maximizing activity outcomes. Dose-response time-kill assays (TKAs) were performed with GSK286 alone and in combination with B and Pa. Monotherapy and combination effects were measured, along with the contribution of each compound and pairwise combination to the overall combination.

## MATERIALS AND METHODS

### Mycobacterial strains and culture conditions

The following Mtb strains were used: Erdman wild-type (wt), H37Rv (GenBank ID: NC_000962.3), Erdman *Rv1625c* overexpressor (OE) strain harboring pMV306/*Rv1625c* recombinant plasmid; Erdman resistant EM19 mutant harboring Ile11fs mutation in the *Rv1625c* gene, streptomycin-starved Mtb 18b (SS18b) strain (11, 12), and the H37Rv-destabilized GFP plasmid (H37Rv-desGFP) (13). The Mtb SS18b strain, a streptomycin-dependent mutant, was used as a model for non-replicating bacteria, while Mtb H37Rv and Mtb Erdman strains were used to represent replicating conditions.

Characterized bacterial stocks of the H37Rv and Erdman strains were prepared in advance by growing cells in 7H9 broth (supplemented with 0.5% glycerol, 10% BBL Middlebrook OADC, and 0.025% tyloxapol) at 37°C to a final optical density at 600 nm (OD_600_) between 0.5 and 0.8. Bacterial stocks were titered by determining the colony-forming units per milliliter (CFU/mL). Aliquots of these characterized stocks were stored at −80°C and thawed to prepare the inoculum for each drug activity experiment.

Non-replicating Mtb SS18b was prepared as follows. The Mtb SS18b strain was expanded in Middlebrook 7H9 broth supplemented with 0.5% glycerol, 0.025% tyloxapol, 10% OADC enrichment, and 50 µg/mL streptomycin at shaking conditions (100 rpm, 37°C). To perform experiments in non-replicating conditions, the strain was starved in acetate medium for 14 days to induce the non-replicating state. Frozen aliquots of the strain were thawed and adjusted to OD = 0.2 and grown in Middlebrook 7H9 broth supplemented with 1 g/L sodium acetate, 0.85 g/L sodium chloride, 5 g/L bovine serum albumin, and 0.05% tyloxapol at shaking conditions (100 rpm, 37°C). Minimum inhibitory concentration (MIC) determination and TKA experiments were performed after 14 days of starvation. CFU counts of inoculum and drug-treated cultures were determined after 6 weeks of growth in Middlebrook 7H10 agar supplemented with 0.5% glycerol, 10% OADC enrichment, and 50 µg/mL streptomycin.

### MIC determination

The extracellular MIC of GSK286 (N70167-41-1, GlaxoSmithKline, GSK) against the Mtb Erdman wt strain was determined using the resazurin microtiter assay (REMA) in seven different broth media: (i) standard broth (ST) 7H9, supplemented with 0.5% glycerol and 10% OADC; (ii) standard broth with tyloxapol: 7H9 supplemented with 10% OADC, 0.5% glycerol, and 0.05% tyloxapol; (iii) acetate broth: 7H9 supplemented with 0.5% BSA, 0.085% NaCl, and 10 mM sodium acetate; (iv) cholesterol (CHO) broth: 7H9 supplemented with 0.5% BSA, 0.085% NaCl, 0.05% tyloxapol, and 0.004% cholesterol (100 µM); (v) fatty acids broth (FA): 7H9 supplemented with 0.5% BSA, 0.085% NaCl + 0.001% palmitic acid, 0.001% stearic acid, and 0.001% oleic acid; (vi) propionate (PRO) broth: 7H9 supplemented with 0.5% BSA, 0.085% NaCl, and 100 µM sodium propionate; (vii) butyrate broth: 7H9 supplemented with 0.5% BSA, 0.085% NaCl, and 5 mM sodium butyrate. The MIC of GSK286 in Mtb *Rv1625c* OE and Mtb Erdman EM19 mutant strains was only tested in CHO and PRO broths. Briefly, 3 days before the start of the experiment, a bacterial suspension with approximately 10^4^ CFU/mL in each broth medium was prepared from a frozen, characterized bacterial stock. This suspension was then incubated at 37°C for 2 days in static conditions to allow bacteria to adapt to the new broth until a final inoculum of approximately 10^5^ CFU/mL before compound addition. The bacterial inoculum was then added to 96-well flat-bottom plates containing 10 twofold serial dilutions of GSK286 diluted in each of the broths listed above (100 μL of bacterial inoculum + 100 µL of compound suspension per well). Plates were wrapped in a plastic bag to prevent evaporation and incubated at 37°C for 7 days. Thirty microliters of a resazurin solution (0.01% wt/vol) (Resazurin powder, reference number R7017-1G, Sigma-Aldrich) in sterile, distilled water were added to each well, and bacterial growth was evaluated by reading the color change and fluorescence (Ex 535 nm/Em 590 nm) using a microplate reader 24 h and 48 h after resazurin addition. The concentration ranges tested for GSK286 and moxifloxacin (MXF) against the Mtb Erdman wt strain were from 0.013 to 26.35 μg/mL and from 0.0002 to 0.5 μg/mL, respectively. Concentration ranges tested for Mtb *Rv1625c* OE and Mtb Erdman EM19 mutant were from 0.004 to 1 μg/mL and from 0.031 to 8 μg/mL, respectively. MIC_50_ and MIC_80_ values are defined as the lowest drug concentration that decreases growth by ≥50% and ≥20%, respectively. Growth percentage was calculated using the following equation:


$$Growth\;\%=\left[\frac{\left(sample\;fluorescence\right)-\left(mean\;negative\;control\right)}{\left(mean\;positive\;control\right)-\left(mean\;negative\;control\right)}\right]\times 100$$

Values were considered valid if Z-score was >0.5 and internal controls performed as expected. MIC values were determined from the first time-point reading with a valid Z-score. MIC determinations were performed at least in two biological replicates with technical triplicates.

Additionally, the activity of GSK286 was evaluated against the Mtb H37Rv strain grown in both standard and CHO broth media, as well as against the non-replicating Mtb SS18b strain cultured in acetate medium and CHO broth media, using REMA and CFU counts. Moxifloxacin was included as an internal control to assess antimicrobial activity against both replicating and non-replicating Mtb.

### Extracellular time-kill assays

TKAs in monotherapy were first performed to assess the extracellular activity of GSK286 alone against Mtb Erdman wt. The following concentrations of GSK286 were tested in CHO and PRO broths: 0.04 µg/mL (1/20× MIC), 0.16 µg/mL (1/5× MIC), 0.41 µg/mL (1/2× MIC), 0.82 µg/mL (1× MIC), 1.65 µg/mL (2× MIC), 4.12 µg/mL (5× MIC), 16.47 µg/mL (20× MIC), and 49.2 µg/mL (60× MIC). Broth media were selected according to the MIC assay results, and the most frequent MIC_80_ value was used to calculate TKA doses. An untreated control for each carbon source broth was also included in the assay. Briefly, a bacterial suspension with approx. 10^4^ CFU/mL (in each broth medium) was initially prepared from a frozen characterized bacterial stock and incubated at 37°C for 3 days in static conditions to let bacteria adapt to the new medium and reach a concentration of approximately 10^5^ CFU/mL before drug treatment. The inoculum culture was then exposed to GSK286 at the above-mentioned concentrations for 50 days at 37°C in a flask format under static conditions. On days 0, 3, 8, 14, 21, 28, 35, and 50, samples were collected and plated using the 2.5 µL plating method in triplicate. Briefly, 10-fold serial dilutions were performed in PBS with 0.1% tyloxapol of each sample. Then, 2.5 µL of the serial dilution samples were transferred to a rectangular plate filled with 7H10 agar with 10% OADC, 0.5% glycerol, and 0.1% tyloxapol for bacterial CFU enumeration (14, 15) and drug efficacy assessment. Agar plates were incubated at 37°C for 21 days, and CFU were enumerated after 14 and 21 days of incubation. CFU/mL were calculated based on the 21 day counts as the average of three replicates and reported as log_10_ CFU/mL.

GSK286 was also evaluated in a separate TKA against Mtb Erdman *Rv1625c* OE (MIC: 0.008 µg/mL–0.016 µg/mL) and Mtb Erdman EM19 mutant (MIC: >8 µg/mL) in CHO broth for 28 days at 37°C, under static conditions. The Mtb Erdman wt strain was included as an internal control. Bacteria were treated with a single dose of GSK286 at 15 µg/mL, which corresponds to 60× MIC against the Mtb Erdman (wild-type) (MIC: 0.25 µg/mL), 937.5× MIC against Mtb Erdman *Rv1625c* OE, and 2× MIC against Mtb Erdman EM19 (mutant) strains. Moxifloxacin 10× MIC (0.6 µg/mL) was used as a control of killing activity. Bacterial survival was assessed by plating diluted samples collected on days 0, 3, 7, 12, 21, and 28 days of incubation on 7H10 agar plates using the 2.5 µL plating method (14, 15).

The activity of GSK286 against the non-replicating Mtb SS18b strain and Mtb H37Rv was also assessed over time in CHO medium. A bacterial inoculum at 10^5^–10^6^ CFU/mL was treated with the compound at concentrations ranging from 0.016 (0.0625× MIC) to 64 µg/mL (256× MIC) in a 96-well plate format. Moxifloxacin was included as a killing activity control. Bacterial survival was assessed by plating diluted samples on 7H10 agar plates on days 0, 7, 14, and 21.

Combinatorial TKAs were conducted to evaluate the *in vitro* activity of GSK286 in combination with bedaquiline (B; reference number FF07153068, TB Alliance) and pretomanid (Pa; reference number 11356-156, TB Alliance) against Mtb H37Rv in CHO broth. Five GSK286 concentrations (0.04, 0.2, 2, 10, and 50 µg/mL) were combined with BPa at matching effective concentrations (EC): EC_5_ (0.058 and 0.053 µg/mL, respectively), EC_10_ (0.103 and 0.105 µg/mL, respectively), and EC_50_ (0.923 and 0.948 µg/mL, respectively). EC_5_, EC_10_, and EC_50_ are concentrations equivalent to 5%, 10%, and 50% of the drug’s maximum efficacy, respectively. TKA included an untreated control, monotherapies, pairwise combinations (B + Pa, GSK286 + B, and GSK286 + Pa), and the three-way combination (B + Pa + GSK286). The efficacy of the compounds/combinations was determined by measuring bacterial growth by CFU enumeration and quantification of the most probable number (MPN) of bacteria at different time points: 0, 3, 7, 14, 21, and 49 days of incubation (14, 15). Hierarchical clustering was conducted to explore similarities among the tested conditions in extracellular TKAs, divided into three subgroups: monotherapies, two-way, and three-way combinations. The optimal clustering method was selected for each subgroup using CFU and MPN data. For CFU, average linkage was used for monotherapies and three-way combinations, while median linkage was applied to two-way combinations. For MPN, average linkage was used for two-way and three-way combinations, and centroid linkage for monotherapies. Euclidean distance was used for clustering rows. Data analysis and visualization were performed in R (version 4.4.2) using the libraries readxl (1.4.3), janitor (2.2.1), pheatmap (1.0.12), magrittr (2.0.3), tidyverse (2.0.0), and RColorBrewer (1.1.3).

### Intracellular time-kill assays

Peripheral blood mononuclear cells (PBMCs) were first isolated from blood samples of healthy human donors by centrifugation through a density gradient medium (Ficoll-Paque Plus, Cytiva). Briefly, blood was diluted in RPMI 1640 (Gibco/Thermo Fisher Scientific) at a 1:1 volume ratio and transferred into a fresh tube containing density gradient medium Ficoll at a 2:1 volume ratio. After centrifugation at 1,600 rpm for 25 min, the interphase was carefully harvested, transferred to a new tube, and washed twice with RPMI 1640 by an initial centrifugation at 1,800 rpm for 10 min, followed by a second centrifugation at 1,000 rpm for 10 min. The pellet was then collected and resuspended in MACS buffer. Subsequently, CD14^+^ monocytes were isolated from the PBMCs by magnetic-activated cell sorting (MACS) technology (Miltenyi). CD14^+^ monocyte-derived macrophages were chosen as they represent a physiologically relevant primary human cell model that closely mimics the macrophage environment encountered by Mtb during infection, in contrast to immortalized cell lines. The pelleted cells were resuspended in MACS buffer and mixed with CD14 magnetic microbeads. The sample was incubated for 10 min at 4°C. MACS buffer was then added, and the cell suspension was centrifuged at 1,000 rpm for 10 min. Magnetic separation was then performed, and cells were seeded in 96-well plates at a concentration of 100,000 cells/well in 100 µL RPMI-FBS-M-CSF (RPMI 1640 medium containing 10% fetal bovine serum [FBS] and 50 ng/mL M-CSF) and incubated for 4 days at 37°C with 5% CO_2_.

The intracellular activity of GSK286 was evaluated against Mtb Erdman wt and the resistant mutant EM19 strains, at concentrations of 1.65 µg/mL and 8.23 µg/mL. The reported intracellular IC_50_ of GSK286 in THP-1 (ATCC TIB-202) cells is 0.02 µg/mL (9). Briefly, 100,000 bacteria in 100 µL (equivalent to a multiplicity of infection [MOI] of 1) were added to the wells containing macrophages and incubated for 3 h at 37°C to allow macrophage infection. To remove extracellular bacteria, wells were washed three times with PBS. Infected cells were treated with DMSO (control) and GSK286 at the two above-mentioned concentrations by adding 100 µL of compound diluted in RPMI-FBS to each well. At given time points (0, 2, 4, 7, and 10 days), 50 µL of 0.1% SDS was added to the wells, mixed, and incubated for 2 min. Ten-fold serial dilutions in PBS were prepared before plating 10 µL onto 7H10 agar plates with 10% OADC. Plates were incubated at 37°C, and CFU were enumerated on days 14 and 21. Data analysis and visualization were performed with R (version 4.3.0) using the ggplot2 (3.5.2), Rmisc (1.5.1), extrafont (0.19), dplyr (1.1.2), ggsignif (0.6.4), ggpubr (0.6.0), and rstatix (0.7.2) packages.

The activity of GSK286 against intracellular Mtb H37Rv was also evaluated in a dose-response assay using THP-1 (ATCC TIB-202) macrophages infected with a GFP-expressing Mtb H37Rv reporter strain. THP-1 macrophages were differentiated with 50 ng/mL phorbol 12-myristate 13-acetate (PMA) for 3 days and then infected with the reporter strain in tissue culture flasks at an MOI of 2 for 2 h. Extracellular bacteria were removed by three washing steps with PBS, and infected cells were seeded into 384-well plates containing GSK286 at a concentration ranging from 0.004 µg/mL to 16 µg/mL and bedaquiline at a concentration ranging from 0.0005 µg/mL to 2 µg/mL. Untreated controls were also included. Intracellular bacterial growth was monitored over time using automated confocal fluorescence microscopy by an InCell 6500 HS high-content imaging system (Cytiva; Molecular Devices). Images were acquired at days 1, 2, 4, 7, and 10 post-treatments and were analyzed using Columbus 2.9.1 Imaging Software (Revvity) with algorithms and scripts described in detail elsewhere (16).

Finally, the intracellular efficacy of the combination BPa-GSK286 against Mtb H37Rv-desGFP was also evaluated in THP-1 cells, routinely cultured in RPMI 1640 medium containing 10% FBS, 1 mM pyruvate, and 2 mM L-glutamine. THP-1 cells were differentiated with 40 ng/mL PMA for 4 h at 37°C with 5% CO_2_, and then infected overnight with H37Rv-desGFP at a MOI of 5. Infected cells were washed, harvested using TrypLE Express Enzyme for cell detachment, and plated onto 384-well plates with pre-dispensed compounds, dissolved in DMSO (final DMSO concentration of 0.5% vol/vol). BDQ at a concentration of 0.0011 µg/mL, PTD at 0.007 µg/mL, and GSK286 at 6.6 µg/mL were tested as monotherapies and in combinations (pairwise and three-way combination). Specific drug concentrations were selected based on previous experiments wherein dose-response curves for each compound individually were characterized. For BDQ and PTD, sub-efficacious doses were deliberately employed (i.e., concentrations that exert minimal effect alone) to allow determination of their individual contributions within the dual and triple combinations. Plates were sealed with breathable plate sealing films, and brightfield and GFP signals were monitored in a continuous manner with an Incucyte SX1 live-cell analysis system (Sartorius).

### Drug stability

The stability of B, Pa, and GSK286 was assessed by LC-MS/MS. For B and Pa, assays were conducted for 7 days in technical duplicates. The compounds were diluted into CHO broth to obtain solutions at 1× MIC (0.12 µg/mL for B and 0.05 µg/mL for Pa) or 10× MIC (1.2 µg/mL for B and 0.5 µg/mL for Pa) and incubated at 37°C for 7 days. Samples were collected at day 0, 1, 4, and 7 and precipitated in 1:10 acetonitrile (AcN). After centrifugation at 12,000 rpm for 10 min at 4°C, samples were transferred into matrix tubes and conserved at −80°C for LC-MS/MS analysis. The LC-MS/MS analysis was performed using a UPLC Acquity I-Class system (Waters) equipped with an Ethylene Bridge Hybrid (BEH) C18 50 × 2.1 mm, 1.7 µm column coupled with a mass spectrometer instrument (Xevo TQ-D system, Waters). The mobile phase consisted of 5 mM ammonium formate in water (phase A) and in AcN (phase B) at a flow rate of 0.6 mL/min, with an elution gradient of 4.0 min from 2% to 98% of phase B and an injection volume of 1 µL. The mass spectrometer equipped with an ESI source was operated in positive ion polarity with a specific MRM method for each compound (transitions 555.19/57.99 for B and 360.11/175.02 for Pa). Raw data were processed within MassLynx software (version 4.2, Waters).

GSK286 stability was assessed using samples collected from TKA monotherapy at concentrations of 16.47 µg/mL and 49.2 µg/mL. Ten microliters of each sample was added to 200 µL of protein precipitant buffer (AcN/methanol 80:20 vol/vol containing the internal standard), then filtered through a 0.45 µm pore size filter before LC-MS/MS analysis. Quantification was performed using UPLC-MS/MS (Waters) system coupled with a Sciex API 4000 instrument (AB Sciex, Toronto, Canada). Filtered samples were loaded onto an Acquity UPLC BEH C18 50 × 2.1 mm, 1.7 µm column (Waters Corporation, Madison, USA) and eluted by gradient with 10 mM ammonium formate plus 0.1% formic acid and AcN at a flow rate of 0.4 mL/min. The MS/MS system was operated in MRM positive mode with a 330.1/280.3 transition.

## RESULTS

### GSK286 is active in cholesterol- and propionate-containing broth media under replicating growth conditions

GSK286 MIC determinations against Mtb Erdman wild-type and derivative strains were performed in various culture conditions with different carbon sources, including: 7H9 supplemented with glycerol, glycerol and tyloxapol, sodium acetate, CHO, fatty acids, sodium PRO, and sodium butyrate. Overall, MIC values inhibiting 80% and 50% of mycobacterial growth (MIC_80_ and MIC_50_, respectively) as determined by REMA varied depending on culture conditions (Table 1; Fig. S1). As expected, no inhibitory activity was found in the presence of glycerol (with and without tyloxapol) (Fig. S1). MIC_50_ values ranging from 0.41 to 0.82 µg/mL were obtained in acetate medium; an MIC_50_ value of 0.41 µg/mL was instead found in fatty acids and sodium butyrate media (Table 1; Fig. S1). In the presence of cholesterol, the MIC_50_ range for GSK286 was between 0.05 and 0.10 µg/mL, while in the presence of propionate, it was 0.05 µg/mL. MIC_80_ values were only obtained in the presence of propionate and cholesterol, ranging from 0.25 to 0.82 µg/mL in CHO and 0.82 µg/mL in PRO (Table 1; Fig. S1). However, lower MIC_80_ values were obtained for the Mtb Erdman *Rv1625c* OE strain: 0.008 to 0.016 μg/mL in CHO and 0.016 to 0.032 μg/mL in PRO. In contrast, the Mtb Erdman EM19 (*Rv1625c* mutant) strain had an MIC_80_ higher than 8 μg/mL in both tested broths. No MIC_90_ was determined in any of the tested conditions (Table 1; Fig. S1). Similar to Mtb Erdman, GSK286 was also active against replicating Mtb H37Rv in CHO medium, but not in regular medium containing OADC and glycerol, while moxifloxacin was active in both types of media (Fig. S2). In contrast, although a sigmoidal dose-response curve for the non-replicating Mtb SS18b strain was observed by REMA, showing inhibition at GSK286 concentrations of 0.125 µg/mL and above, this activity was not confirmed by CFU counts. No significant reduction in CFU/mL was detected between untreated controls and samples treated with the highest GSK286 concentration (64 µg/mL), regardless of whether bacteria were cultured in cholesterol or in regular starvation medium containing acetate (Fig. S2B). The expected activity of moxifloxacin, used as an assay control, against replicating (Mtb H37Rv) and non-replicating (Mtb SS18b) strains was observed (Fig. S1B).

**TABLE 1:** *In vitro* activity of GSK286 against different Mtb Erdman strains in liquid medium containing various carbon sources^*a*^

Carbon sources	Mtb Erdman wild-type		Mtb Erdman *Rv1625c* OE	Mtb Erdman EM19 (*Rv1625c* mutant)
	MIC_50_ (µg/mL)	MIC_80_ (µg/mL)	MIC_80_ (µg/mL)	MIC_80_ (µg/mL)
Standard glycerol	ND	ND	NA	NA
Standard glycerol with tyloxapol	ND	ND	NA	NA
Cholesterol	0.10	0.21–0.82	0.008–0.016	>8
Propionate	0.21	0.82	0.032	>8
Acetate	0.41	ND	NA	NA
Fatty acids	0.41	ND	NA	NA
Butyrate	0.41	ND	NA	NA

^*a*^ ND, not determined; out of the tested range of concentrations. NA, not applicable. OE overexpressing.

### GSK286 exhibits limited bacteriostatic activity against Mtb strains

**Fig 1:**
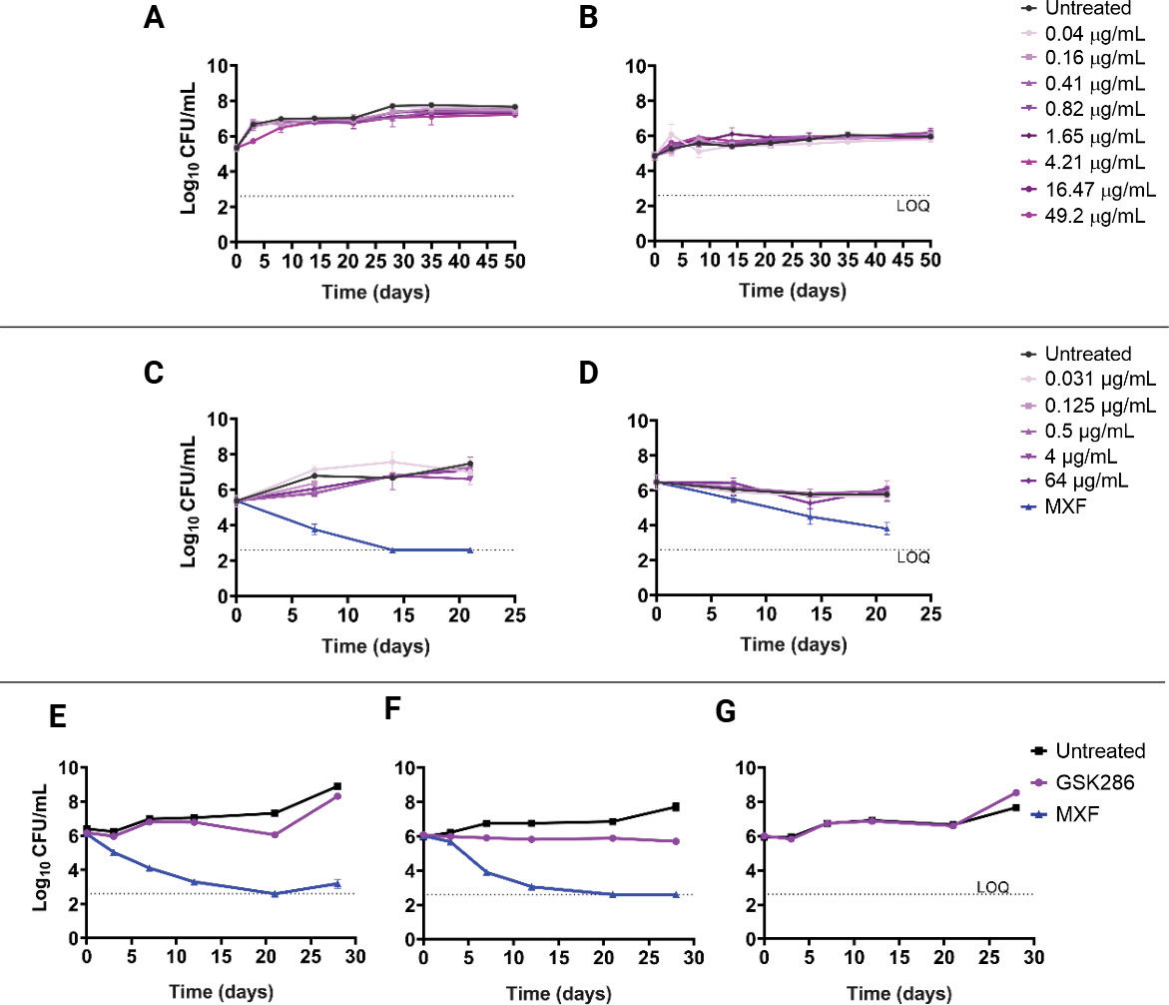
Extracellular activity of GSK286 against Mtb. Dose-response TKA of GSK286 in monotherapy. Mtb Erdman wild-type exposed to a concentration range of GSK286 for 50 days at 37°C in (**A**) cholesterol and (**B**) propionate media. (**C**) Mtb H37Rv and (**D**) Mtb SS18b (non-replicating) strains treated with a concentration range of GSK286 and MXF at 8× MIC (2 µg/mL) in cholesterol medium. (**E**) Mtb Erdman wild-type, (**F**) Mtb Erdman *Rv1625c* OE (overexpressing strain), and (**G**) Mtb Erdman EM19 (*Rv1625c* mutant) strains exposed to a single dose of GSK286 at 15 µg/mL and MXF at 10× MIC (0.6 µg/mL), as a positive control in cholesterol medium. The mean of log_10_ CFU/mL replicate samples is plotted. LOQ, limit of quantification; MXF, moxifloxacin.

Dose-response TKAs of GSK286 monotherapy did not allow for EC50 calculations of the dose response of the compound. However, they showed slight growth delay activity of the compound against extracellular Mtb Erdman wild-type in cholesterol broth, but not in propionate, at early time points (up to day 10) with the highest concentration (ca. 50 µg/mL) (Fig. 1A and B). However, this was not observed with the Mtb H37Rv and non-replicating SS18b strains over a 21 day treatment period (Fig. 1C and D). When comparing *Rv1625c*-derivative strains, GSK286 exhibited clear bacteriostatic activity against the overexpressing *Rv1625c* strain, with CFU counts remaining close to the inoculum bacterial concentration over time, while the EM19 (*Rv1625c* mutant) strain was resistant to GSK286, growing similarly to the untreated control (Fig. 1E through G). These data support the role of the *Rv1625c* gene in GSK286 *in vitro* activity.

**Fig 2:**
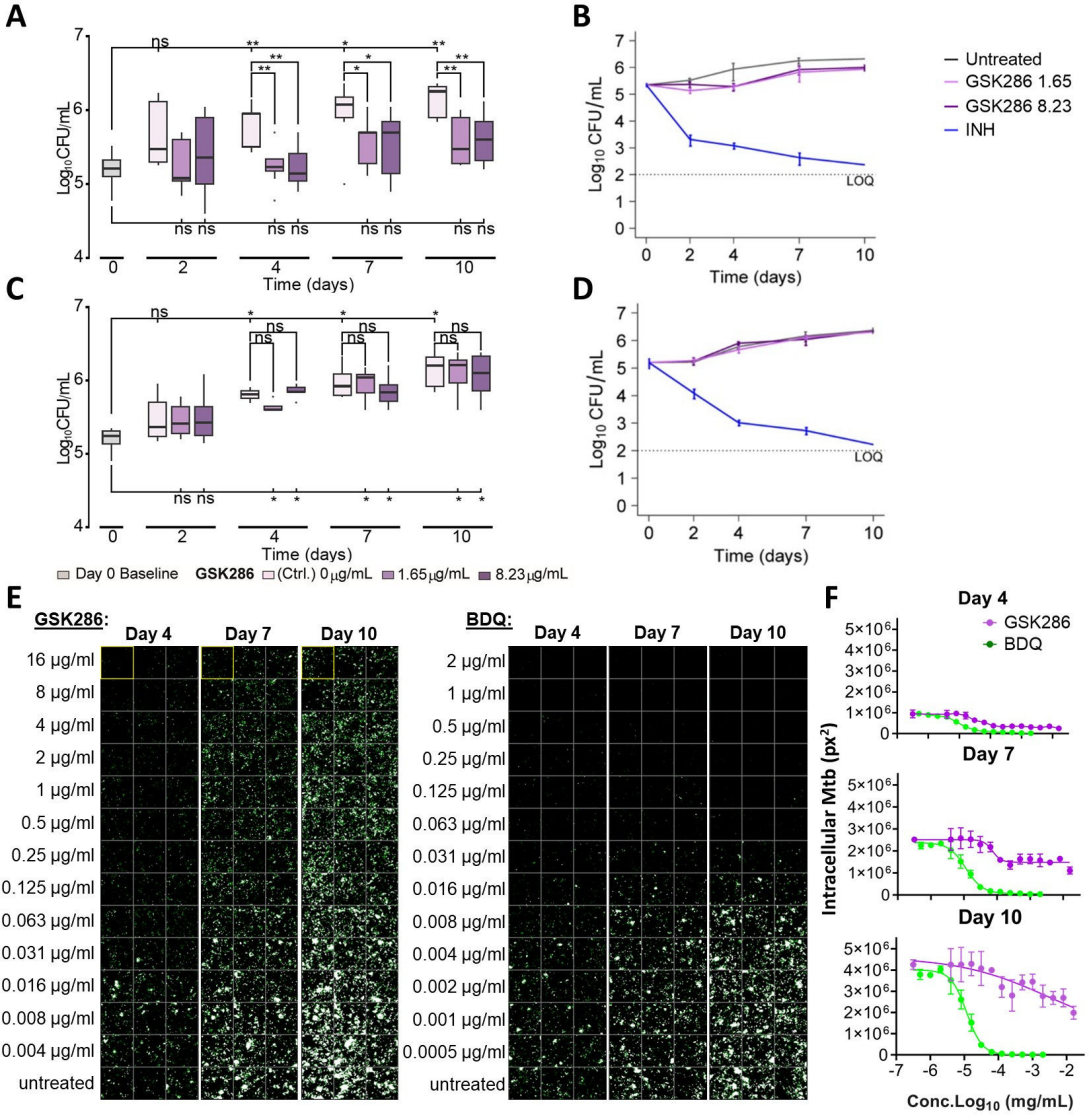
Intracellular activity of GSK286 against Mtb. The activity of GSK286 at 1.65 µg/mL and 8.23 µg/mL against the Mtb Erdman wild-type (**A and B**) and Mtb EM19 (resistant *Rv1625c* mutant) (**C and D**) strains was measured intracellularly in infected macrophages derived from human CD14^+^ monocytes over a period of 10 days. Two-way ANOVA and *post hoc* Wilcoxon test (non-parametric) with Benjamini-Hochberg correction were performed for statistical analyses. ns, not significant, * = *P* < 0.05; ** = *P* < 0.01. (**E**) Confocal microscopy images at days 4, 7, and 10 post-treatment of intracellular Mtb H37Rv-GFP infecting THP-1 macrophages and treated with increasing concentrations of GSK286. BDQ is used as a positive control. (**F**) Quantification of the bacterial burden based on pixel intensity (px²) relative to log_10_ drug concentration at days 4, 7, and 10 post-treatment.

The intracellular activity of GSK286 was evaluated by CFU enumeration against Mtb Erdman wild-type and EM19 (*Rv1625c* mutant) strains in infected macrophages derived from human CD14^+^ monocytes and treated for 10 days. Similar to extracellular conditions, GSK286 exhibited a bacteriostatic effect against the wild-type (but not the resistant mutant, as expected), with growth inhibition observed at concentrations of 1.65 µg/mL and 8.23 µg/mL up to day 4, after which growth resumed (Fig. 2A through D). A similar pattern was observed against intracellular H37Rv-GFP in THP-1 macrophages, using confocal fluorescence microscopy (Fig. 2E and F).

### GSK286 shows *in vitro* concentration-dependent interaction with the BPa backbone

**Fig 3:**
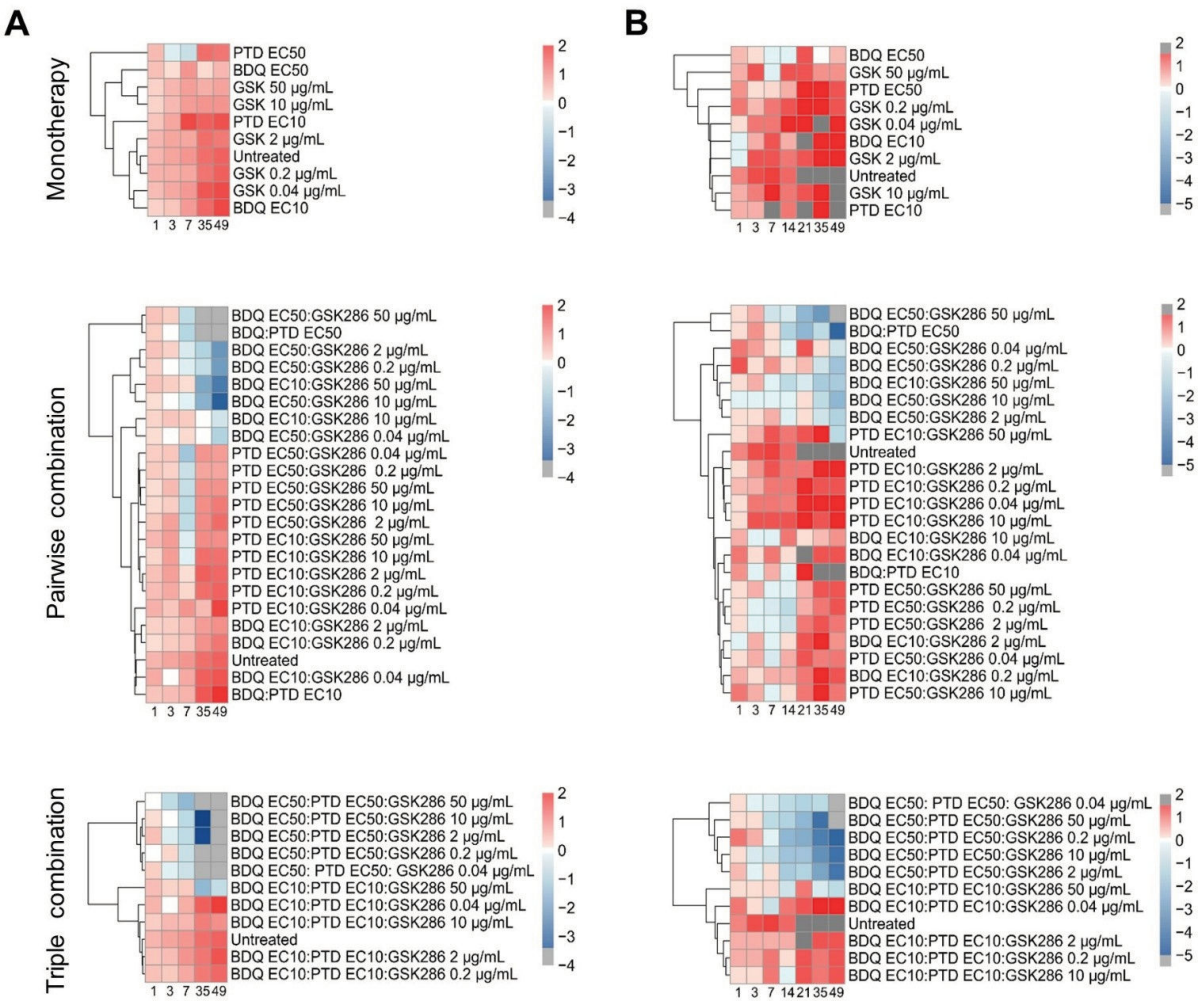
Combinatorial studies of GSK286 with bedaquiline and pretomanid. Time-kill assay heat map representation of CFU (**A**) and MPN (**B**) counts over 50 days of treatment with GSK286, BDQ, and PTD in monotherapy, pairwise, and triple combination against Mtb H37Rv. Columns represent selected time points (days), and rows represent tested conditions. Cell values represent log_10_ CFU/mL relative to the untreated inoculum at day 0. Red indicates growth. Blue shades indicate bacterial burden reduction, with darker shades representing stronger effects. Light gray represents BLQ (CFU < 400 CFU/ml, MPN < 150 MPN/mL) and darker gray ALQ (MPN > 5.5 × 10^8^ MPN/mL). CFU, colony-forming unit; MPN, most probable number; BDQ, bedaquiline; PTD, pretomanid; BLQ, below limit of quantification; ALQ, above limit of quantification; EC, effective concentration. EC_10_: 0.103 µg/mL and 0.105 µg/mL and EC_50_: 0.923 µg/mL and 0.948 µg/mL for B and Pa, respectively.

**Fig 4:**
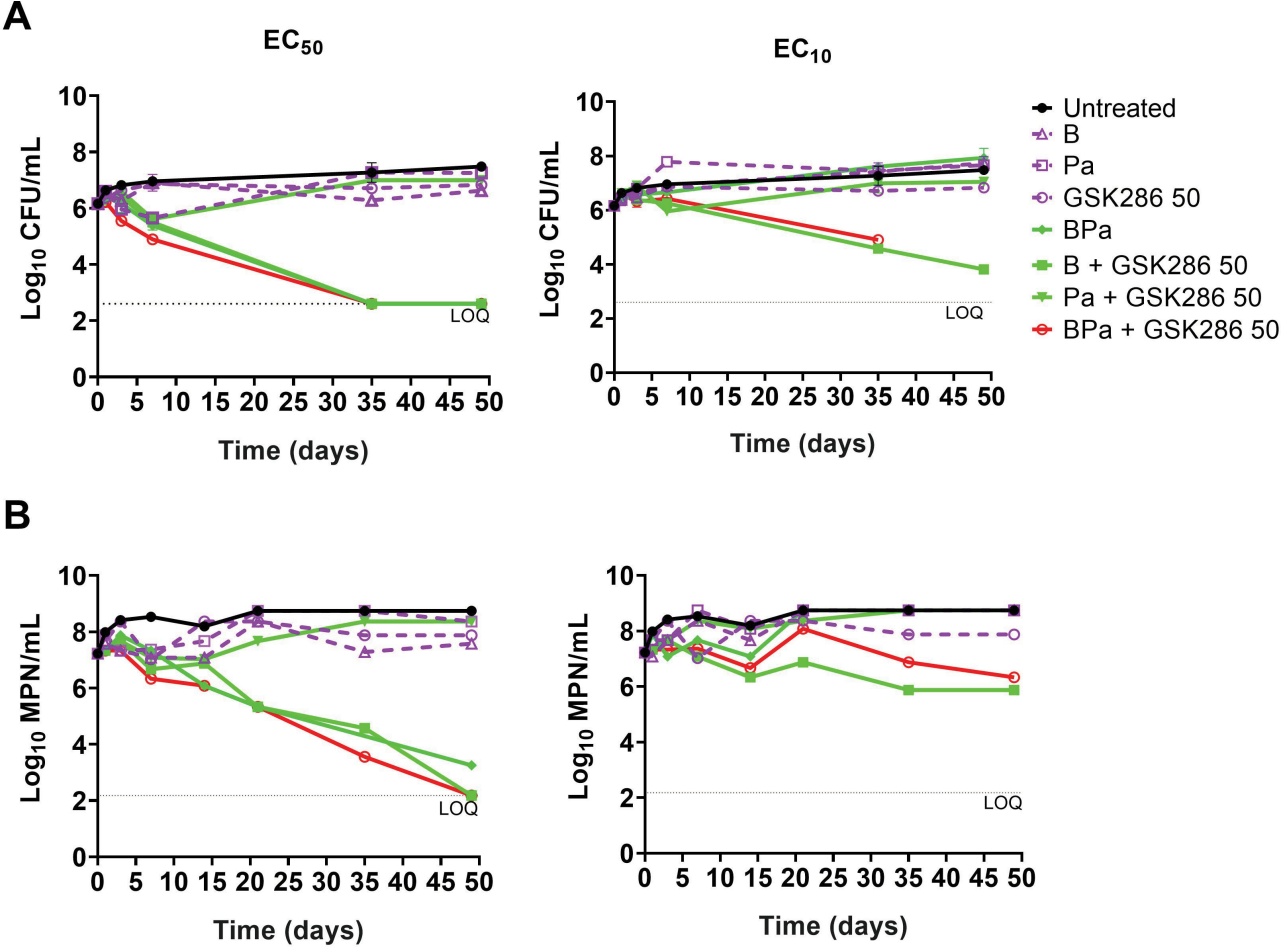
Kill kinetics of GSK286 with bedaquiline and pretomanid in extracellular conditions. Time-kill curves of Mtb H37Rv treated with GSK286 (50 µg/mL) combined with BPa at their matching EC_50_ and EC_10_. (**A**) CFU-based data. (**B**) MPN-based data. Data from triple, pairwise combinations and monotherapies are shown. The mean log_10_ CFU/mL from triplicate samples and log_10_ MPN/mL measured at different time points are plotted. EC_10_: 0.103 µg/mL and 0.105 µg/mL and EC_50_: 0.923 µg/mL and 0.948 µg/mL for B and Pa, respectively. B, bedaquiline. Pa, pretomanid. LOQ, limit of quantification.

The activity of GSK286, B, and Pa was evaluated individually, in pairwise, and three-way combinations under extracellular conditions against Mtb H37Rv in CHO broth, using CFU enumerations and MPN calculations. Data from these assays are summarized in heat maps (Fig. 3), while time-kill curves demonstrating the dynamic effects of the combinations are presented in Fig. 4.

Overall, the contribution of GSK286, B, and Pa was dependent on the concentrations used for each drug in the combination (Fig. 3). When evaluated as monotherapy, individual drugs did not have an inhibitory effect on their own at the tested concentrations (upper panels of Fig. 3). These concentrations were purposely selected to have a sub-inhibitory effect; however, the overall efficacy improved when drugs were applied in combination. The BPa-GSK286 combination exhibited its highest observed killing only when B and Pa were administered at their EC50, independently of the concentration used of GSK286. However, this interaction was mainly governed by the pairwise BPa backbone (Fig. 3 and 4). Lowering the concentrations of B and Pa to their EC10 values revealed that GSK286 at 50 µg/mL exhibited the strongest interaction when combined with B, leading to a greater bacterial burden reduction than the BPa backbone, and achieving efficacy comparable to the triple BPa-GSK286 combination (Fig. 3 and 4). Reducing GSK286 concentration to 10 µg/mL allowed the combination with BDQ to effectively inhibit bacterial growth but without complete bacterial eradication (Fig. 3; Fig. S4). When GSK286 was tested at concentrations lower than 10 µg/mL, there was no discernible interaction or effect observed (Fig. 3; Fig. S5 to S7).

Similarly, when B and Pa concentrations were reduced to EC5, the combination of GSK286 (50 µg/mL) and B was more active than the triple combination (BPa-GSK286) or even the BPa backbone (Fig. S8).

**Fig 5:**
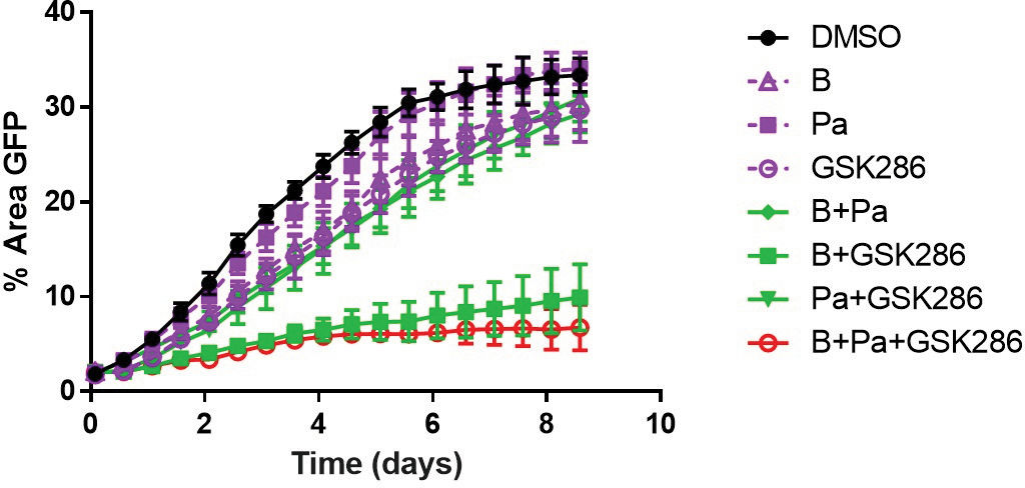
Activity kinetics of GSK286 with bedaquiline and pretomanid in intracellular conditions. Dose-response curves of GSK286, B, and Pa in monotherapy and in combination against intracellular Mtb H37Rv-desGFP in THP-1 cells. The graph represents the percentage area of GFP signals continuously monitored over 9 days using the Incucyte live-cell analysis system as a readout. B was tested at a concentration of 0.011 µg/mL, Pa at 0.007 µg/mL, and GSK286 at 6.6 µg/mL.

GSK286 has been previously described to have intracellular activity (10). In our study, similar patterns of interaction were observed in intracellular conditions. The combination of GSK286 and B showed stronger interaction than the BPa backbone and achieved potencies comparable to the triple BPa-GSK286 combination. As expected, lower concentrations were needed to observe such effects in intracellular conditions (Fig. 5), compared to the extracellular activity (Fig. 4).

Quantitative analysis of B, Pa, and GSK286 by mass spectrometry at different concentrations demonstrated stability of all three compounds *in vitro* in cholesterol medium at 37°C tested up to 7 days of incubation. At day 7, and depending on the tested concentration, more than 83% of all three compounds were still present in the tested medium (Table S1).

## DISCUSSION

The activity of GSK286 under various *in vitro* culture conditions and its inhibitory activity against Mtb was media-specific. As expected, GSK286 was not active in standard 7H9 broth media, while it inhibited Mtb growth in cholesterol-containing media and, notably, also in the presence of propionate. This may align with GSK286’s proposed mechanism of action, which targets Mtb by disrupting cholesterol metabolism through modulation of the Mtb adenylyl cyclase Rv1625c. This triggers excessive cyclic AMP (cAMP) production in a *Rv1625c*-dependent manner that disrupts cholesterol utilization, a crucial carbon source for Mtb during infection (9). Given that propionate is a major by-product of cholesterol degradation and that cAMP signaling is also implicated in propionate metabolism in Mtb (17, 18), it is plausible that GSK286 interferes with propionate metabolism pathways in a similar manner. However, direct evidence linking GSK286 to impaired propionate degradation in Mtb remains hypothetical.

Although the REMA methodology is widely used to assess antimicrobial efficacy, this study demonstrated discrepancies between REMA and CFU methods for GSK286, with REMA not accurately reflecting GSK286’s actual activity. A reduction in the REMA signal was observed with the non-replicating SS18b strain in acetate and cholesterol media, which suggests that GSK286 is effective against non-replicating Mtb. A similar strong inhibitory effect of the compound was observed against Mtb in cholesterol medium. However, these reductions did not correlate with CFU counts, which showed no reduction in the growth of non-replicating bacteria and minimal inhibition of Mtb (Fig. S2). This discrepancy can be explained by GSK286’s mechanism of action, which involves blocking cholesterol degradation (9). This impairs bacterial respiration and energy metabolism without necessarily affecting bacterial viability. Therefore, since REMA is based on the reduction of resazurin by cellular oxidoreductases, the observed signal reduction likely reflects metabolic disruption rather than true bactericidal or bacteriostatic effects. These findings underscore the importance of validating metabolic activity assays with viability measurements, such as CFU enumeration, when interpreting the activity of GSK286 *in vitro*.

Further supporting the role of Rv1625c in mediating GSK286 activity, our results showed that the MIC of GSK286 was markedly reduced in a bacterial strain overexpressing *Rv1625c*, compared to the wild-type strain (Table 1). Conversely, the Mtb mutant with a non-functional *Rv1625c* gene exhibited resistance to GSK286. These findings align with previous studies demonstrating that overexpression of *Rv1625c* increases Mtb susceptibility to GSK286 in the presence of cholesterol (Fig. 1) (9).

To better explore the full activity of GSK286 both in monotherapy and in combination, we conducted a dose-response TKA extending over 50 days, whereas most *in vitro* preclinical evaluations with Mtb are typically performed only over a short-term 14- to 21-day period (14). To our knowledge, this is the first time the activity of GSK286 has been evaluated in an extended TKA. Short-term TKAs are sufficient for assessing the early bactericidal effects observed within the first days to weeks of treatment. However, they often fail to capture delayed responses, bacterial regrowth, or the emergence of resistance. Extending assay duration more accurately reflects the chronic nature of TB infection and offers a full spectrum of drug activity over time, including long-term sterilizing potential and potential bacterial regrowth dynamics. Additionally, this approach is particularly relevant when evaluating novel compounds or drugs with a delayed onset of action, such as BDQ, where increased killing or sterilizing effects may only emerge after prolonged exposure (19). In our extended TKA, we also integrated an additional readout, the MPN, alongside the conventional CFU enumeration used in preclinical *in vitro* studies. MPN is a culture-based method that estimates bacterial counts by detecting their growth in liquid media, similar to the time-to-positivity readout used in clinical trials. As a liquid-based method, it offers greater sensitivity, particularly at low bacterial loads, and can detect viable but non-culturable subpopulations that are overlooked by the CFU method (20, 21). In line with this, our data showed that MPN consistently yielded higher estimates of bacterial counts than CFU enumeration. For instance, while CFU counts showed apparent sterilization by day 35 for the combination of GSK286 and BPa, the MPN method detected surviving bacteria until day 49 (Fig. 3 and 4). This finding highlights the added value of integrating additional biomarkers to enable a more comprehensive assessment of bacterial viability and culture sterilization, thereby enhancing the accuracy of *in vitro* drug evaluation.

When tested in monotherapy, GSK286 demonstrated minimal antibacterial activity against both extracellular and intracellular Mtb (Fig. 1 and 2; Fig. S2) throughout the entire experiment. Quantitative analysis confirmed that the compound remained chemically stable by 7 days (> 80% drug recovered) (Table S1), thereby excluding degradation or loss of potency as potential reasons for its lack of efficacy at early time points. Although GSK286 alone did not significantly reduce Mtb viability, it strongly contributes to enhanced bacterial killing in combination therapy with the BPa backbone.

Findings from the extracellular *in vitro* evaluation demonstrated concentration-dependent activity of the triple combination. This achieved highest observed killing at EC_50_ of B and Pa, an effect primarily governed by the BPa backbone. Under these conditions, varying the concentration of GSK286 did not alter the outcome, suggesting that the use of EC_50_ may reduce the dynamic range to observe the contributions of GSK286. When the backbone concentration was reduced, this became more evident. With B at EC_10_, GSK286 at 50 µg/mL enhanced the activity of B, producing a greater killing effect than the BPa backbone alone and achieving efficacy comparable to the triple BPa-GSK286 combination.

Findings from this *in vitro* study also revealed that the contribution of GSK286 is dose-dependent, with its strongest effect observed at higher concentrations when the backbone is administered below EC_50_. This dose-response relationship underscores the need to consider clinically relevant exposures and pharmacokinetic-pharmacodynamic (PK-PD) modeling to determine whether the observed conditional contributions translate into therapeutic benefit. Furthermore, the confirmed stability of B and Pa during the assay substantiates that the bactericidal effects observed are attributable to the drugs’ actions and interactions on the bacteria, rather than experimental artifacts. In the case of GSK286 (>83% of stability at day 7), if we assume a constant decrease rate due to drug degradation in a 50 day TKA, this would result in a significant drug concentration loss at the endpoint. Nonetheless, an inhibitory effect is still observed for the combination of GSK286 with BPa, thus suggesting a possible post-antibiotic effect. In this sense, our study provides *in vitro* confirmation of the contribution of GSK286 when combined with B and Pa (Fig. 3 and 4; Fig. S3 through S6), a regimen previously shown to be effective in *in vivo* studies (10).

Importantly, this strong interaction between B and GSK286 observed under extracellular conditions was further confirmed under intracellular conditions (Fig. 5), reinforcing the potential of this dual combination in targeting intracellular bacteria. These findings align with the *in vivo* data reported by Nuermberger et al., which demonstrated that GSK286 enhances BPa activity (10).

Another notable observation is that GSK286 showed enhanced potency against intracellular bacteria, requiring lower concentrations to achieve comparable effects to those in extracellular cholesterol-based conditions (Fig. 2 and 5). These observations are supported by previous data reporting that the concentration required to inhibit 50% of bacterial growth (IC_50_) was markedly lower in THP-1 macrophages, measured at 0.07 µM (0.02 µg/mL), than in axenic culture at 0.7 µM (0.2 µg/mL) (10). This 10-fold difference in potency underscores the greater efficacy of GSK286 within the host-cell environment and it is consistent with *in vivo* studies using BALB/c mice, in which nearly all bacilli reside intracellularly, and GSK286 exhibited bactericidal activity at a relatively lower dose (≥1.38 µg/mL C_max_) (10). Interestingly, a similar intracellular enhancement was observed for B and Pa: significantly lower concentrations of these drugs (0.011 µg/mL and 0.007 µg/mL for B and Pa, respectively) were sufficient to achieve comparable effects to those seen in extracellular conditions in combination assays (0.103 µg/mL and 0.105 µg/mL for B and Pa, respectively) (Fig. 5). It is known that the intracellular activity of GSK286 is linked to cAMP production (9). Understanding the molecular basis of the enhanced potency of the BPa-GSK286 combination would require further investigation beyond the scope of this study. Although GSK286 demonstrated better potency under intracellular conditions, such CFU-based assays are not well suited to high-throughput evaluation, necessitating the use of extracellular conditions for broader evaluation. Additionally, other intracellular methodologies, such as confocal microscopy or IncuCyte imaging used in this study, reflect inhibitory activity but do not directly measure bactericidal effects, only measured by CFU- and MPN-based assays; thus, necessitating a combination of different methodologies to fully characterize the potential of GSK286-based combinations.

While there is solid evidence supporting the role of GSK286 in enhancing BPa anti-TB activity, our *in vitro* data emphasize the specific favorable contribution of GSK286 in combination with B, particularly under conditions of reduced B doses. Using lower concentrations of B may be advantageous in mitigating side effects while maintaining treatment effectiveness; similarly, it could enhance the activity of B in low-level resistant settings mediated by *Rv0678* mutations. Considering evidence of lower Pa activity in L1 (4, 5) and the emergence of Pa-resistant clinical isolates of Mtb (6), the combination of B + GSK286 could be explored as an alternative backbone for the L1 lineage. This situation stresses the importance of relying on multiple treatment options for diverse disease scenarios, including the exploration of nitroimidazole alternatives. This concept is further supported by a recent report showing that the combination of GSK286 + B + TBA7371 is as effective as BPaL in murine models, when analyzing bactericidal and sterilizing activities (22). Therefore, reinforcing the idea that the replacement of Pa with GSK286 (or other CHO-dependent compounds) in current anti-TB/MDR-Rif-R regimens could represent an attractive alternative. In fact, several CHO-dependent compounds are currently under development (23).

A limitation of the present study, previously discussed, is that all experiments described herein were performed with lineage 4 based strains. As already mentioned, there is evidence showing that the activity of Pa appears to vary by MTBC lineage, with L1 intrinsically less susceptible than other major lineages. This raises the question of whether BPaL regimen remains equally effective in patients infected with this lineage, representing approximately 28% of all global TB cases (4, 5). Moreover, in the TKA experimental set-up, there is no concentration modulation across the assay. Instead, a single drug dosage is administered at the beginning of each experiment, which does not allow PK mimicking modulation studies. Future studies should contemplate the use of dynamic PK-PD models such as the hollow-fiber system for tuberculosis (HFS-TB). The HFS-TB is a preclinical tool endorsed by the European Medicines Agency for the study of Mtb drug treatments that provides dynamic measures of pharmacokinetic and pharmacodynamic relationships of antimicrobial treatments against a pathogen of interest (24, 25).

In summary, our results provide a comprehensive understanding of the complexity and concentration dependencies of GSK286’s interactions with B and Pa, and highlight the translational potential of *in vitro* assays. Moreover, they provide valuable insights into the potential synergies and limitations of these drug combinations, paving the way for further investigations and tailored approaches in TB treatment strategies. These data are particularly valuable for translational modeling frameworks, as they enable the generation of a comprehensive distribution of interaction terms among drugs, which could aid the identification of optimized drug combinations and dosages for a more effective and improved treatment against Mtb. Future studies integrating PK-PD modeling to simulate clinically relevant exposures will be critical to refine our understanding of the dynamic interplay among agents in the BPa-GSK286 (or CHO-dependent compounds) combination and to optimize combination strategies.

## SUPPLEMENTAL MATERIAL

Supplemental material aac.00260-26-s0001.docx Table S1; Fig. S1 to S8.

ASM does not own the copyrights to Supplemental Material that may be linked to, or accessed through, an article. The authors have granted ASM a non-exclusive, world-wide license to publish the Supplemental Material files. Please contact the corresponding author directly for reuse.

## References

[1] World Health Organization. 2025. Global tuberculosis report. Geneva, Switzerland

[2] WHO consolidated guidelines on tuberculosis. Module 4: treatment – drug-resistant tuberculosis treatment, 2022 update. 2022. World Health Organization. Geneva, Switzerland. Available from: https://iris.who.int/handle/10665/365308 36630546

[3] Conradie F, Bagdasaryan TR, Borisov S, Howell P, Mikiashvili L, Ngubane N, Samoilova A, Skornykova S, Tudor E, Variava E, et al.. 2022. Bedaquiline-pretomanid-linezolid regimens for drug-resistant tuberculosis. N Engl J Med 387:810–823. doi:10.1056/NEJMoa2119430 36053506 PMC9490302

[4] Rupasinghe P, Reenaers R, Vereecken J, Mulders W, Cogneau S, Merker M, Niemann S, Vally Omar S, Rigouts L, Köser CU, Decroo T, de Jong BC. 2024. Refined understanding of the impact of the Mycobacterium tuberculosis complex diversity on the intrinsic susceptibility to pretomanid. Microbiol Spectr 12:e0007024. doi:10.1128/spectrum.00070-24 38334384 PMC10913522

[5] Bateson A, Ortiz Canseco J, McHugh TD, Witney AA, Feuerriegel S, Merker M, Kohl TA, Utpatel C, Niemann S, Andres S, et al.. 2022. Ancient and recent differences in the intrinsic susceptibility of Mycobacterium tuberculosis complex to pretomanid. J Antimicrob Chemother 77:1685–1693. doi:10.1093/jac/dkac070 35260883 PMC9155602

[6] Goig GA, Windels EM, Loiseau C, Stritt C, Biru L, Borrell S, Brites D, Gagneux S. 2025. Ecology, global diversity and evolutionary mechanisms in the Mycobacterium tuberculosis complex. Nat Rev Microbiol 23:602–614. doi:10.1038/s41579-025-01159-w 40133503

[7] Netikul T, Palittapongarnpim P, Thawornwattana Y, Plitphonganphim S. 2021. Estimation of the global burden of Mycobacterium tuberculosis lineage 1. Infect Genet Evol 91:104802. doi:10.1016/j.meegid.2021.104802 33684570

[8] VanderVen BC, Fahey RJ, Lee W, Liu Y, Abramovitch RB, Memmott C, Crowe AM, Eltis LD, Perola E, Deininger DD, Wang T, Locher CP, Russell DG. 2015. Novel inhibitors of cholesterol degradation in Mycobacterium tuberculosis reveal how the bacterium’s metabolism is constrained by the intracellular environment. PLoS Pathog 11:e1004679. doi:10.1371/journal.ppat.1004679 25675247 PMC4335503

[9] Brown KL, Wilburn KM, Montague CR, Grigg JC, Sanz O, Pérez-Herrán E, Barros D, Ballell L, VanderVen BC, Eltis LD. 2023. Cyclic AMP-mediated inhibition of cholesterol catabolism in Mycobacterium tuberculosis by the novel drug candidate GSK2556286. Antimicrob Agents Chemother 67:e0129422. doi:10.1128/aac.01294-22 36602336 PMC9872607

[10] Nuermberger EL, Martínez-Martínez MS, Sanz O, Urones B, Esquivias J, Soni H, Tasneen R, Tyagi S, Li S-Y, Converse PJ, et al.. 2022. GSK2556286 is a novel antitubercular drug candidate effective in vivo with the potential to shorten tuberculosis treatment. Antimicrob Agents Chemother 66:e0013222. doi:10.1128/aac.00132-22 35607978 PMC9211396

[11] Hashimoto T. 1955. Experimental studies on the mechanism of infection and immunity in tuberculosis from the analytical standpoint of streptomycin-dependent tubercle bacilli. 1. Isolation and biological characteristics of a streptomycin-dependent mutant, and effect of streptomycin administration on its pathogenicity in guinea-pigs. Kekkaku 30:4–8. 14354794

[12] Sala C, Dhar N, Hartkoorn RC, Zhang M, Ha YH, Schneider P, Cole ST. 2010. Simple model for testing drugs against nonreplicating Mycobacterium tuberculosis. Antimicrob Agents Chemother 54:4150–4158. doi:10.1128/AAC.00821-10 20679505 PMC2944619

[13] Manina G, Dhar N, McKinney JD. 2015. Stress and host immunity amplify Mycobacterium tuberculosis phenotypic heterogeneity and induce nongrowing metabolically active forms. Cell Host Microbe 17:32–46. doi:10.1016/j.chom.2014.11.016 25543231

[14] van Wijk RC, Lucía A, Sudhakar PK, Sonnenkalb L, Gaudin C, Hoffmann E, Dremierre B, Aguilar-Ayala DA, Molin MD, Rybniker J, de Giorgi S, Cioetto-Mazzabò L, Segafreddo G, Manganelli R, Degiacomi G, Recchia D, Pasca MR, Simonsson USH, Ramón-García S, ERA4TB consortium. 2023. Implementing best practices on data generation and reporting of Mycobacterium tuberculosis in vitro assays within the ERA4TB consortium. iScience 26:106411. doi:10.1016/j.isci.2023.106411 37091238 PMC10119593

[15] Rabodoarivelo MS, Hoffmann E, Gaudin C, Aguilar-Ayala DA, Galizia J, Sonnenkalb L, Dal Molin M, Cioetto-Mazzabò L, Degiacomi G, Recchia D, Rybniker J, Manganelli R, Pasca MR, Ramón-García S, Lucía A, ERA4TB consortium. 2025. Protocol to quantify bacterial burden in time-kill assays using colony-forming units and most probable number readouts for Mycobacterium tuberculosis. STAR Protoc 6:103643. doi:10.1016/j.xpro.2025.103643 40067825 PMC11931380

[16] Deboosere N, Belhaouane I, Machelart A, Hoffmann E, Vandeputte A, Brodin P. 2021. High-content analysis monitoring intracellular trafficking and replication of Mycobacterium tuberculosis inside host cells. Methods Mol Biol 2314:649–702. doi:10.1007/978-1-0716-1460-0_29 34235675

[17] Hayden JD, Brown LR, Gunawardena HP, Perkowski EF, Chen X, Braunstein M. 2013. Reversible acetylation regulates acetate and propionate metabolism in Mycobacterium smegmatis. Microbiology (Reading) 159:1986–1999. doi:10.1099/mic.0.068585-0 23813678 PMC3783017

[18] Nambi S, Gupta K, Bhattacharyya M, Ramakrishnan P, Ravikumar V, Siddiqui N, Thomas AT, Visweswariah SS. 2013. Cyclic AMP-dependent protein lysine acylation in mycobacteria regulates fatty acid and propionate metabolism. J Biol Chem 288:14114–14124. doi:10.1074/jbc.M113.463992 23553634 PMC3656268

[19] Koul A, Vranckx L, Dhar N, Göhlmann HWH, Özdemir E, Neefs J-M, Schulz M, Lu P, Mørtz E, McKinney JD, Andries K, Bald D. 2014. Delayed bactericidal response of Mycobacterium tuberculosis to bedaquiline involves remodelling of bacterial metabolism. Nat Commun 5:3369. doi:10.1038/ncomms4369 24569628 PMC3948051

[20] Chengalroyen MD, Beukes GM, Gordhan BG, Streicher EM, Churchyard G, Hafner R, Warren R, Otwombe K, Martinson N, Kana BD. 2016. Detection and quantification of differentially culturable tubercle bacteria in sputum from patients with tuberculosis. Am J Respir Crit Care Med 194:1532–1540. doi:10.1164/rccm.201604-0769OC 27387272 PMC5215032

[21] Hu Y, Liu A, Ortega-Muro F, Alameda-Martin L, Mitchison D, Coates A. 2015. High-dose rifampicin kills persisters, shortens treatment duration, and reduces relapse rate in vitro and in vivo. Front Microbiol 6:641. doi:10.3389/fmicb.2015.00641 26157437 PMC4477163

[22] Li SY, Tyagi S, Soni H, Betoudji F, Converse PJ, Mdluli K, Upton AM, Fotouhi N, Barros-Aguirre D, Ballell L, Jimenez-Navarro E, Nuermberger EL. 2024. Bactericidal and sterilizing activity of novel regimens combining bedaquiline or TBAJ-587 with GSK2556286 and TBA-7371 in a mouse model of tuberculosis. Antimicrob Agents Chemother 68:e0156223. doi:10.1128/aac.01562-23 38376228 PMC10989019

[23] Wilburn KM, Montague CR, Qin B, Woods AK, Love MS, McNamara CW, Schultz PG, Southard TL, Huang L, Petrassi HM, VanderVen BC. 2022. Pharmacological and genetic activation of cAMP synthesis disrupts cholesterol utilization in Mycobacterium tuberculosis. PLoS Pathog 18:e1009862. doi:10.1371/journal.ppat.1009862 35134095 PMC8856561

[24] Cavaleri M, Manolis E. 2015. Hollow fiber system model for tuberculosis: the European medicines agency experience. Clin Infect Dis 61 Suppl 1:S1–S4. doi:10.1093/cid/civ484 26224766

[25] Aguilar-Ayala DA, Sanz-García F, Rabodoarivelo MS, Susanto BO, Bailo R, Eveque-Mourroux MR, Willand N, Simonsson USH, Ramón-García S, Lucía A, ERA4TB consortium. 2024. Evaluation of critical parameters in the hollow-fibre system for tuberculosis: a case study of moxifloxacin. Br J Clin Pharmacol 90:1711–1727. doi:10.1111/bcp.16068 38632083

