# Supplementary material for: In vitro characterization of GSK2556286 in combination with bedaquiline and pretomanid against Mycobacterium tuberculosis
Source: Antimicrob Agents Chemother. 2026 Jul 13;70(8):e00260-26. doi: 10.1128/aac.00260-26 (PMC13436017; doi:10.1128/aac.00260-26)
Supplement: Supplementary file 1 [file aac.00260-26-s0001.docx]

**SUPPLEMENTARY INFORMATION**

***In vitro* characterization of GSK2556286 in combination with Bedaquiline and Pretomanid against *Mycobacterium tuberculosis***

Marie-Sylvianne Rabodoarivelo^1,†,*^, Jordana Galizia^1,†^, Deborah Recchia^2^, Alessandro Stamilla^2^, Michael Dal Molin^3,4^, Eik Hoffmann^5^, Cyril Gaudin^5^, Maxime Eveque^6^, Dominique Ndjogou^6^, Rebeca Bailo^1^, Albin A.M. Leding^7^, Ulrika S.H. Simonsson^7^, Olalla Sanz^8^, Elena Jiménez^8^, Robert Bates^8^, Giulia Degiacomi^2^, Ainhoa Lucía^1,9^, Nicolas Willand^6^, Alain Baulard^5^, Jan Rybniker^3,4,10^, Maria Rosalia Pasca^2,11^, Santiago Ramón-García^1,9,12,*^, on behalf of the ERA4TB consortium.

^1^ Department of Microbiology, Pediatrics, Radiology and Public Health, Faculty of Medicine, University of Zaragoza, Zaragoza, Spain;

^2^ Department of Biology and Biotechnology Lazzaro Spallanzani, University of Pavia, Pavia, Italy;

^3^ Department I of Internal Medicine, Division of Infectious Diseases, University of Cologne, Cologne, Germany;

^4^ Faculty of Medicine, Center for Molecular Medicine Cologne (CMMC), University of Cologne, Cologne, Germany;

^5^ Institute Pasteur Lille, CNRS, Inserm, UMR9017 / U1019 - Center for Infection and Immunity of Lille (CIIL), Lille, France;

^6^ Univ. Lille, Institute Pasteur Lille, Inserm, U1177 - Drugs and Molecules for Living Systems, Lille, France;

^7^ Department of Pharmaceutical Biosciences, Uppsala University, Uppsala, Sweden;

^8^ Global Health Medicines R&D, GlaxoSmithKline GSK, Tres Cantos, 28760 Madrid, Spain;

^9^ CIBER Enfermedades Respiratorias (CIBERES), Instituto de Salud Carlos III, Madrid, Spain;

^10^ German Center for Infection Research (DZIF), Partner Site Bonn-Cologne, Cologne, Germany;

^11^ Fondazione IRCCS Policlinico San Matteo, Pavia, Italy;

^12^ Research and Development Agency of Aragon Foundation (Fundación ARAID), Zaragoza, Spain;

^†^Equal contribution

# *Corresponding authors: Marie Sylvianne Rabodoarivelo: [mrabodoarivelo@unizar.es](mailto:mrabodoarivelo@unizar.es) and Santiago Ramón-García: [santiramon@unizar.es](mailto:santiramon@unizar.es)

This work has received support from the Innovative Medicines Initiatives 2 Joint Undertaking (grant No 853989)

**Table S1. Stability of compounds in cholesterol medium.** GSK286, bedaquiline and pretomanid were incubated at different concentrations in cholesterol medium and incubated at 37 °C up to 7 days. Drug quantification was performed by LC-MS/MS. The table shows the percentage of compound remaining, calculated from the measured concentrations.

| Compound | Concentration |  | % remaining at | | | | |
| --- | --- | --- | --- | --- | --- | --- | --- |
|  |  |  | Day 0 | Day 1 | Day 2 | Day 4 | Day 7 |
| GSK286 | 49.2 µg/mL |  |  |  |  |  | **>83** |
| Bedaquiline | 0.12 µg/mL | repl. 1 | 100 | 87 | 91 | 93 | 85 |
|  |  | repl. 2 | 100 | 97 | 92 | 100 | 102 |
|  |  | average | **100** | **92** | **92** | **96** | **93** |
|  | 1.2 µg/mL | repl. 1 | 100 | 99 | 93 | 89 | 84 |
|  |  | repl. 2 | 100 | 99 | 92 | 92 | 90 |
|  |  | average | **100** | **99** | **92** | **90** | **87** |
| Pretomanid | 0.05 µg/mL | repl. 1 | 100 | 101 | 101 | 105 | 97 |
|  |  | repl. 2 | 100 | 90 | 98 | 93 | 92 |
|  |  | average | **100** | **96** | **100** | **99** | **95** |
|  | 0.5 µg/mL | repl. 1 | 100 | 101 | 100 | 100 | 99 |
|  |  | repl. 2 | 100 | 101 | 100 | 99 | 97 |
|  |  | average | **100** | **101** | **100** | **100** | **98** |

**Figure S1. Effect of carbon sources on the activity of GSK286 determined by REMA assay.** Dose-response curves of Mtb Erdman wt across distinct carbon sources. Each curve shows % bacterial growth over a Log_10_ concentration range (µg/mL). Individual replicate (n = 3) are displayed. Shaded regions mark the MIC_80_ threshold, defined as the lowest concentration achieving ≥80% growth inhibition. % Growth was calculated by normalizing REMA RFU values to the mean of untreated control wells.

**Figure S2. Extracellular activity of GSK286 in non-replicating (Mtb SS18b) and replicating (Mtb H37Rv) conditions.** Dose-response curves of Mtb SS18b and H37Rv strains measured by REMA (black symbols, left axis) and CFU plating (green symbols, right axis) of selected conditions treated with either (**A**) moxifloxacin (MXF) or (**B**) GSK286 for 7 days.

**Figure S3. Kill kinetics of GSK286, bedaquiline and pretomanid.** Time-kill curves of the Mtb H37Rv treated with GSK286 alone at different concentrations (0.04, 0.2, 2, 10 and 50 µg/mL), and bedaquiline (B) and pretomanid (Pa) at their EC_10_ and EC_50_ in cholesterol broth. Data was extracted from the combinatorial TKA. (**A**) CFU-based data. (**B**) MPN-based data. The mean log_10_ CFU/mL from triplicate samples and log_10_ MPN/mL measured at different time-point are plotted. EC_10_: 0.103 and 0.105 µg/mL and EC_50_: 0.923 and 0.948 µg/mL for B and Pa, respectively.

**Figure S4. Kill kinetics of bedaquiline and pretomanid in combination (EC_10_ and EC_50_) with GSK286 at 10 µg/mL.** Time-kill curves of Mtb H37Rv treated with GSK286 at 10 µg/mL combined with BPa at their matching EC_50_ and EC_10._ (**A**) CFU-based data. (**B**) MPN-based data. Data from pairwise combinations is also shown. The mean log_10_ CFU/mL from triplicate samples and log_10_ MPN/mL measured at different time-point are plotted. EC_10_: 0.103 µg/mL and 0.105 µg/mL and EC_50_: 0.923 µg/mL and 0.948 µg/mL for B and Pa, respectively. B: Bedaquiline. Pa: Pretomanid. LOQ: Limit of quantification.

**Figure S5. Kill kinetics of bedaquiline and pretomanid in combination (EC_10_ and EC_50_) with GSK286 at 2 µg/mL.** Time-kill curves of Mtb H37Rv treated with GSK286 at 2 µg/mL combined with BPa at their matching EC_50_ and EC_10_. (**A**) CFU-based data. (**B**) MPN-based data. Data from pairwise combinations is also shown. The mean log_10_ CFU/mL from triplicate samples and log_10_ MPN/mL measured at different time-point are plotted. EC_10_: 0.103 µg/mL and 0.105 µg/mL and EC_50_: 0.923 µg/mL and 0.948 µg/mL for B and Pa, respectively. B: Bedaquiline. Pa: Pretomanid. LOQ: Limit of quantification.

**Figure S6. Kill kinetics of bedaquiline and pretomanid in combination (EC_10_ and EC_50_) with GSK286 at 0.2 µg/mL.** Time-kill curves of Mtb H37Rv treated with GSK286 at 0.2 µg/mL combined with BPa at their matching EC_50_ and EC_10_. (**A**) CFU-based data. (**B**) MPN-based data. Data from pairwise combinations is also shown. The mean log_10_ CFU/mL from triplicate samples and log_10_ MPN/mL measured at different time-point are plotted. EC_10_: 0.103 µg/mL and 0.105 µg/mL and EC_50_: 0.923 µg/mL and 0.948 µg/mL for B and Pa, respectively. B: Bedaquiline. Pa: Pretomanid. LOQ: Limit of quantification.

**Figure S7. Kill kinetics of bedaquiline and pretomanid in combination (EC_10_ and EC_50_) with GSK286 at 0.04 µg/mL.** Time-kill curves of Mtb H37Rv treated with GSK286 at 0.04 µg/mL combined with BPa at their matching EC_50_ and EC_10_. (**A**) CFU-based data. (**B**) MPN-based data. Data from pairwise combinations is also shown. The mean log_10_ CFU/mL from triplicate samples and log_10_ MPN/mL measured at different time-point are plotted. EC_10_: 0.103 µg/mL and 0.105 µg/mL and EC_50_: 0.923 µg/mL and 0.948 µg/mL for B and Pa, respectively. B: Bedaquiline. Pa: Pretomanid. LOQ: Limit of quantification.

**Figure S8. Kill kinetics of bedaquiline and pretomanid in combination (EC_5_) with GSK286 at 50 µg/mL.** Time-kill curves of Mtb H37Rv treated with GSK286 at 50 µg/mL combined with BPa at their matching EC_5_. (**A**) CFU-based data and (**B**) MPN-based data. Data from pairwise combinations is also shown. The mean log_10_ CFU/mL from triplicate samples and log_10_ MPN/mL measured at different time-point are plotted. EC_5_: 0.058 µg/mL and 0.053 µg/mL for B and Pa, respectively. B: Bedaquiline. Pa: Pretomanid. LOQ: Limit of quantification.
